# Back-Propagation Operation for Analog Neural Network Hardware with Synapse Components Having Hysteresis Characteristics

**DOI:** 10.1371/journal.pone.0112659

**Published:** 2014-11-13

**Authors:** Michihito Ueda, Yu Nishitani, Yukihiro Kaneko, Atsushi Omote

**Affiliations:** Advanced Research Division, Panasonic Corporation, Soraku, Kyoto, Japan; University of South Australia, Australia

## Abstract

To realize an analog artificial neural network hardware, the circuit element for synapse function is important because the number of synapse elements is much larger than that of neuron elements. One of the candidates for this synapse element is a ferroelectric memristor. This device functions as a voltage controllable variable resistor, which can be applied to a synapse weight. However, its conductance shows hysteresis characteristics and dispersion to the input voltage. Therefore, the conductance values vary according to the history of the height and the width of the applied pulse voltage. Due to the difficulty of controlling the accurate conductance, it is not easy to apply the back-propagation learning algorithm to the neural network hardware having memristor synapses. To solve this problem, we proposed and simulated a learning operation procedure as follows. Employing a weight perturbation technique, we derived the error change. When the error reduced, the next pulse voltage was updated according to the back-propagation learning algorithm. If the error increased the amplitude of the next voltage pulse was set in such way as to cause similar memristor conductance but in the opposite voltage scanning direction. By this operation, we could eliminate the hysteresis and confirmed that the simulation of the learning operation converged. We also adopted conductance dispersion numerically in the simulation. We examined the probability that the error decreased to a designated value within a predetermined loop number. The ferroelectric has the characteristics that the magnitude of polarization does not become smaller when voltages having the same polarity are applied. These characteristics greatly improved the probability even if the learning rate was small, if the magnitude of the dispersion is adequate. Because the dispersion of analog circuit elements is inevitable, this learning operation procedure is useful for analog neural network hardware.

## Introduction

The artificial neural network (ANN) is receiving research interest, for example, due to deep learning approaches that are improving recognition rates in benchmark classification problems [1], [2]. There have been studies on large-scale digital processing built upon the conventional CPUs and GPUs[3]. However, built only with digital circuits [4], the ANN hardware requires a large volume of memory. It is true that the algorithm improvement can reduce the memory size, but cannot solve the fundamental problem. A hardware-level solution must be proposed.

One of the solutions is introducing a neuromorphic device. To realize ANN hardware, the circuit element for synapse function is important because the number of synapse elements is much larger than that of neuron elements. One of the candidates for this synapse element is a memristor [5], [6]. Because the conductance of the memristor depends on the history of the applied voltage, it can realize the synapse function [7], [8]. The memristor-based memories can achieve a very high integration density of 100 Gbit/cm^2^, a few times higher than flash memory technologies [9]. These unique properties make it a promising device for massively parallel, large-scale neuromorphic systems [7], [10]. Hu *et al*. have also reported the potential of a memristor crossbar array that functions as an associative memory [11].

We have also examined the synapse function using a ferroelectric memristor (FeMEM) [12], [13]. Because FeMEM could be operated at a 60 nm channel length [14], high density integration of FeMEM synapse device can be expected. We demonstrated the conductance change according to the biologically inspired learning method of spike-timing-dependent synaptic plasticity (STDP) [15], [16]. As the FeMEM has three terminals, concurrent learning can be realized. We constructed an analog circuit with FeMEM synapses for a Hopfield neural network, and by using this STDP learning method, we demonstrated the learning and recalling of patterns [17], [18].

To realize generic ANN hardware, we should adapt the learning method to a back-propagation (BP) algorithm [19]. Ishii *et al*. reported hardware BP learning for neuron metal–oxide–semiconductor (MOS) neural networks [20]. However, the neuron MOS did not have non-volatile memory to store the learned synapse weight.

By applying a memristor as a multivalued memory, many researchers have reported ANN hardware having memristor synapses [6]–[8], [11], [17], [18], [21]. However, a memristor has hysteresis characteristics of input voltage and conductance. The conductance values vary according to the history of the applied voltage and its width. These characteristics make it difficult to control its conductance to the desired value. Therefore, it is not easy to apply the BP learning algorithm to ANN hardware having memristor synapses. The purpose of this paper is to develop a simple procedure for the BP learning operation, which can be applied to analog ANN hardware with synapse devices having hysteresis and variability.

## Analog Neural Network Hardware with FeMEMs

### 1. Feed-forward neural network

**Figure 1:**
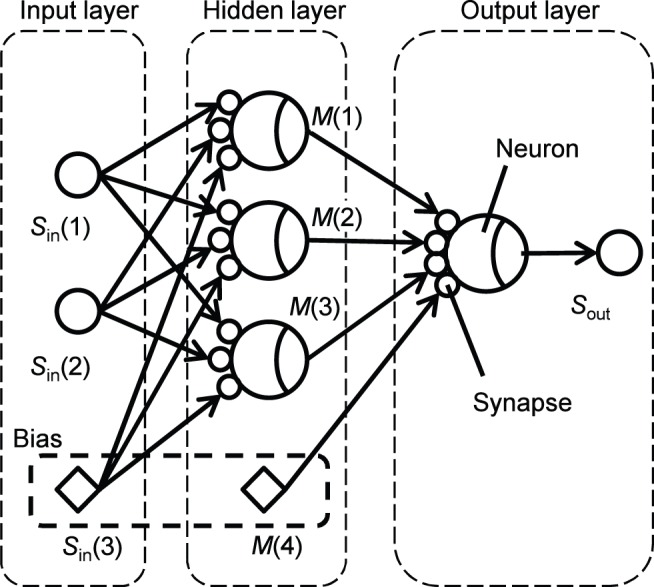
Analyzed feed forward neural network. The structure has two inputs in the input layer, three neurons in the hidden layer, and one neuron in the output layer. *S*_in_(1) and *S*_in_(2) are the inputs. *M*(1)–*M*(3) and *S*_out_ are the output from hidden and output layers, respectively. *S*_in_(3) and *M*(4) are the bias inputs.



Figure 1 shows the analyzed feed-forward neural network structure. This structure has two inputs in the input layer, three neurons in the hidden layer, and one neuron in the output-layer. A neuron has multiple synapses. A fundamental calculation for neural networks is the product sum operation described by

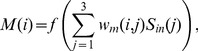
(1)

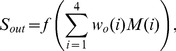
(2)where *M*(*i*) is the output from hidden-layer neurons, *S*_out_ is the output from the output-layer neuron, *S*_in_(1) and *S*_in_(2) are two inputs, *w*_m_(*i*, *j*) and *w*_o_(*i*) are the synapse weights of the hidden-layer neurons and the output-layer neurons, respectively. The function f(–) is a threshold function; a sigmoidal function is frequently used. In Figure 1, both *S*_in_(3) and *M*(4) are bias inputs, and their values are unity.

### 2. ANN hardware

**Figure 2:**
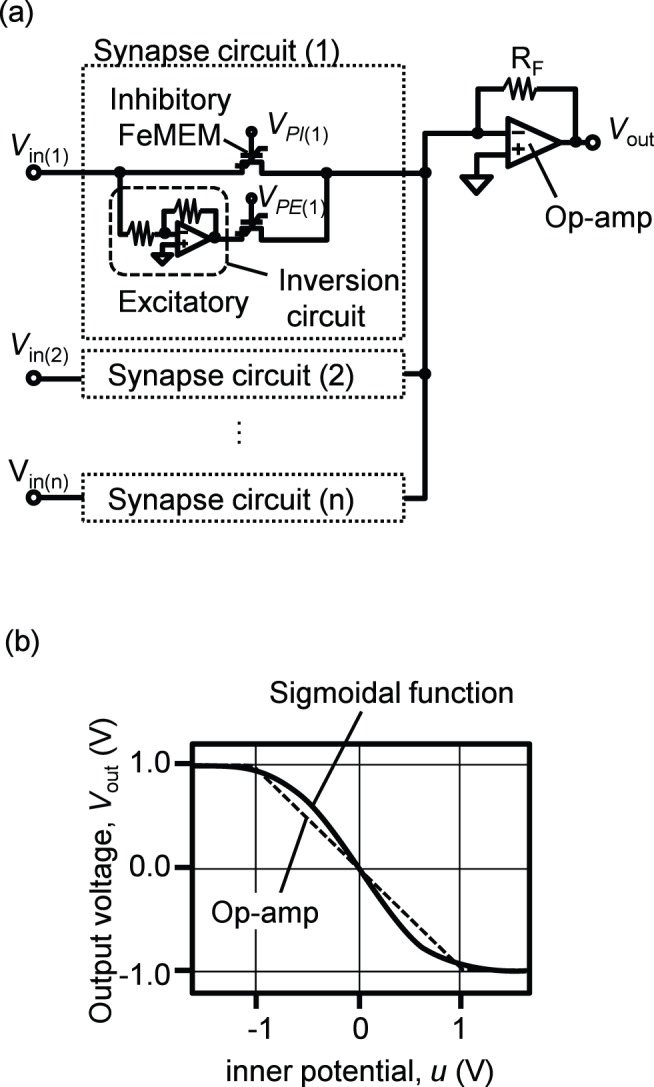
Schematic of a neuron circuit and its input-output characteristics. The neuron circuit is based on an op-amp adder circuit as shown in (a). Synapse circuits are constructed with a FeMEM. To realize positive and negative synapse weights, we adopted excitatory and inhibitory synapses. The inner potential (*u*) is calculated according to (3). The relation between *u* and output voltage (*V*_out_) of the op-amp resembles the input-output characteristics of a sigmoidal function as shown in (b).



To realize an analog neuron device, we examined a circuit based on an operational amplifier (op-amp) adder circuit. Using FeMEMs and an op-amp, a neuron circuit was constructed as shown in Figure 2(a). R_F_ is a fixed resistance, whose conductance is *G*_R_. To achieve a synapse function using an FeMEM, the synaptic circuit modules were devised that consist of inhibitory/excitatory synapse pairs. As the op-amp adder circuit is an inverting amplifier circuit, the inhibitory pairs receive raw input directly and the excitatory ones receive inverted copies of the raw input voltage via a unity gain inverting amplifier. Although this synapse circuit construction needs two FeMEMs, a highly functional neuron circuit can be realized, because the modulation of synapse weight is easier to control individually with two FeMEMs. Here, we denote the channel conductance of FeMEM as *G*_F_. Also, we denote *G*_F_ for the excitatory synapse as *G*_E_(*i*) and *G*_F_ for the inhibitory synapse as *G*_I_(*i*). The sum of amplified voltages, or the inner potential (*u*), is calculated as

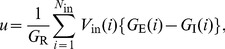
(3)where *N*_in_ is the total number of inputs, *V*_in_(*i*) is the input voltage. The non-linear output voltage (*V*_out_) of the op-amp is



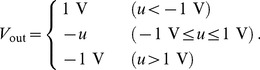
(4)As this circuit is an inverting amplifier circuit, the plus and minus signs of *u* are reversed for the output voltage. Thus, the input voltage for excitatory FeMEM is reversed by using an inversion circuit. Using *G*_F_, the synapse weight (*w*) can be calculated as *G*_F_/*G*_R_. We use the output of the op-amp as that of a neuron circuit for convenience in constructing the circuit, although, in general neural networks, the output of a neuron is calculated using a threshold function such as a sigmoidal function. Figure 2(b) compares a sigmoidal function and the op-amp output. The change in the op-amp output is linear in the voltage range of the power supply and is constant out of the range. Though the linear property of the op-amp may degrade the learning ability, the sigmoidal function and op-amp output have similar trends.

## Preparation of ANN Hardware and Proposal of Learning Operation

### 1. Structure and procedure for preparation of the FeMEM

**Figure 3:**
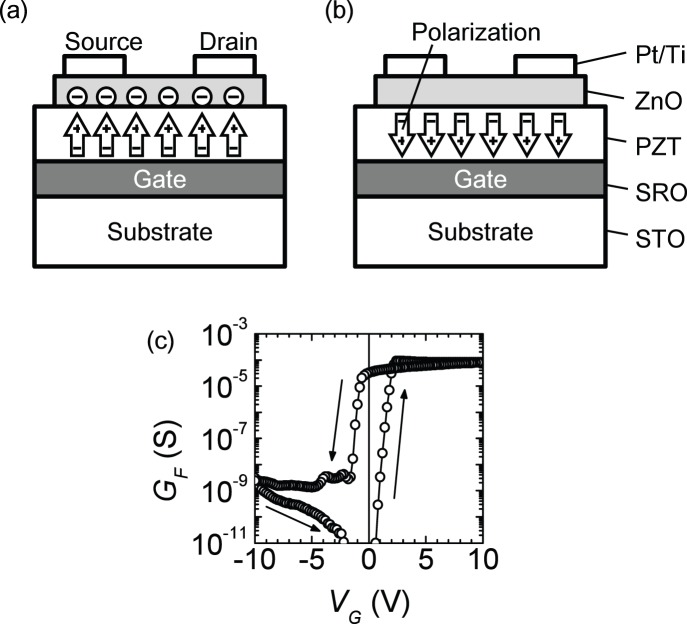
Schematics of FeMEM and its electrical properties. The fabricated FeMEM showed (a) electron gas accumulation and (b) complete depletion switching operation due to the reversal of ferroelectric polarization. (c) *G*_F_–*V_G_* characteristics showed the counterclockwise hysteresis loop corresponding to the switching of ferroelectric polarization.

We fabricated a FeMEM structure based on insights gained in previous studies [12]–[14]. As shown in Figure 3(a) (b), the FeMEM consists of a semiconductor film of ZnO, a ferroelectric film of Pb(Zr,Ti)O_3_ (PZT), and a bottom gate electrode of SrRuO_3_ (SRO). All the layers of ZnO/PZT/SRO were epitaxially grown over a SrTiO_3_ (STO) substrate by pulsed laser deposition. Pt/Ti electrodes were used for the source and drain contacts to the ZnO film. The fabricated FeMEM showed electron gas accumulation and complete depletion switching operation due to reversal of the ferroelectric polarization. The channel conductance (*G*_F_)–gate voltage (*V*_G_) characteristics of the FeMEM are shown in Figure 3(c). The *G*_F_–*V*_G_ characteristics were measured using a semiconductor parametric analyzer (Agilent 4155C) under the condition of long integration time. By measuring the drain current under the condition of drain voltage = 0.1 V, *G*_F_ was calculated. The drain voltage was set to be low so as not to change the polarization of the ferroelectric. The figure shows counterclockwise hysteresis loops corresponding to the switching of ferroelectric polarization. The conductance at *V*_G_ = 0 V changed according to the history of applied *V*_G_ and could thus take multiple values. It was confirmed that there was no notable degradation of conductance over 10^5 ^s [12]. These characteristics allowed the construction of an analog ANN circuit with synapse elements using the FeMEM [15]–[18], [21].

### 2. Electrical characteristics of the synapse circuit

**Figure 4:**
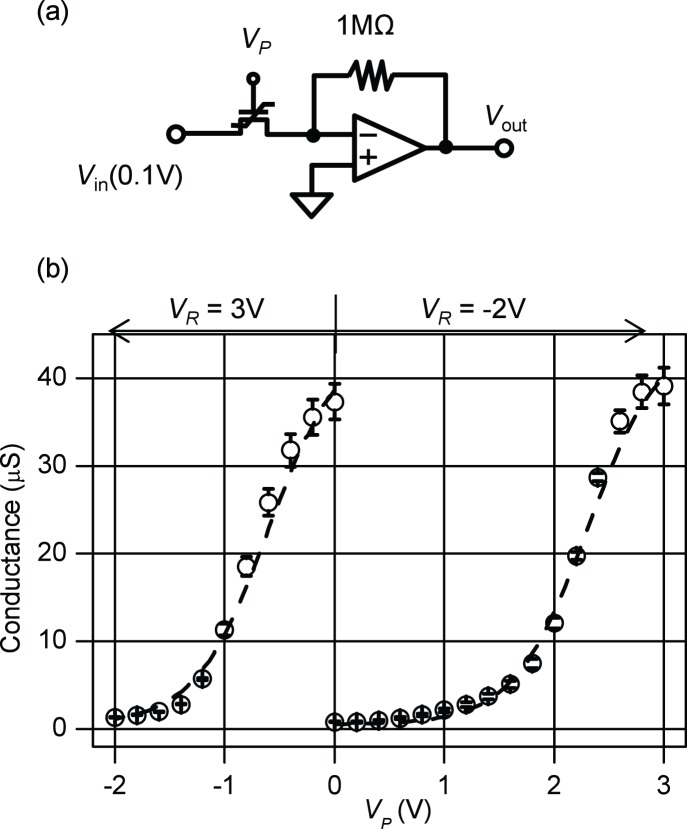
Schematics of measurement setup and calculated conductance. With the measurement setup shown in (a), the conductance of the FeMEM can be calculated from the measured output voltage (*V*_out_) from the op-amp when input voltage *V*_in_ = 0.1 V. After applying a reset pulse (*V*_R_) and a write pulse (*V*_P_) to the gate electrode of the FeMEM, *V*_out_ is measured. The calculated conductance is shown in (b). The open circles indicate the average values and the error bars indicate the standard deviation over 300 scans.

We examined the performance of the basic neuron circuit. The experimental setup used to evaluate the relation of the pulse voltage (*V*_P_) and the conductance of the FeMEM is shown in Figure 4(a). The devices we use in this experiment have been tested before and were found to exhibit good non-volatility characteristics [12]. The pulse width of *V*_P_ was set to 1 ms. To enhance the conductance repeatability, the conductance was measured after applying a reset pulse (*V*_R_). *V*_R_ = −2 V when *V*_P_>0 and *V*_R_ = 3 V when *V*_P_<0. *V*_P_ was first increased from 0 to 3 V in 0.2 V steps and then reduced from 0 to −2 V in −0.2 V steps. In the same manner as *G*_F_–*V*_G_ measurement, the drain current was measured under the condition of drain voltage = 0.1 so as not to change the polarization of the ferroelectric.

This scanning operation was performed 300 times. From the measured *V*_out_, *G*_F_ was calculated according to
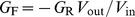
(5)


Because *V*_R_ and *V*_P_ are pulse voltages, the voltages are applied only during the weight update. This enables the reduction of the number of voltage sources as the pulse voltages can be applied by switching a voltage source. Moreover, the power consumption for maintaining the synapse weight is zero.

The average and standard deviation of calculated conductance are shown in Figure 4(b). Smooth counterclockwise characteristics were observed. The conductance change was in the range of 0.5×10^−6^–40×10^−6^ S for the investigated *V*_P_ range.

To analyze a learning operation, as an alternative approach, we prepared a numerical model of the FeMEM conductance by fitting experimental data. We fitted the average conductance with sigmoidal functions that are commonly used in modeling ferroelectric functions [22]. We manually fitted the two curves of increasing voltage and decreasing voltage and derived the equation.
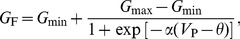
(6)where *α* = 3 V^−1^, *G*_max_ = 45×10^−6^ S, *G*_min_ = 0.5×10^−6^ S, *θ* ( = *θ*_1_) = 2.3 V (for increasing *V*_P_) and *θ* ( = *θ*_2_) = −0.6 V (for decreasing *V*_P_). The fitting curves are shown in Figure 4(b) as broken lines. Approximate characteristics were expressed, regardless of there being few parameters.

### 3. Proposal of BP operation of hysteresis synapse devices

BP is the most widely applied learning methods for training an ANN. When a synapse weight (*w*) is changed, the outputs of the ANN also change, which is known as weight perturbation [23]. The synapse weight *w* is updated according to
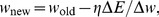
(7)where *η* is the learning rate, *w*_new_ and *w*_old_ are the synapse weights after and before the update, respectively. The square error (*E*) is calculated according to



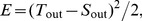
(8)where *T*_out_ is a target output. Because the ANN in this study has only one output, *E* can be calculated simply according to (8). Δ*E* is the difference between the square errors after and before the update and is calculated according to
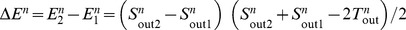
(9)where the subscript 1 and 2 indicates before and after the update, and the superscript *n* indicates the input pattern number defined in table 1.

**Table 1:** Definition of the input pattern number *n*.

n	S_in_(1)	S_in_(2)
1	1 V	1 V
2	1 V	−1 V
3	−1 V	1 V
4	−1 V	−1 V

As the synapse weight (*w*) of the analog ANN hardware in this paper is calculated as *w* = *G*_F_/*G*_R_, *w* is proportional to *G*_F_. Because *G*_F_ is a function of *V*_P_ as shown in (6), we simply updated *V*_P_ according to
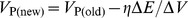
(10)where *V*_P(new)_ and *V*_P(old)_ are *V*_P_s after and before the update, respectively, and Δ*V* is the minute *V*_P_ change to obtain the error difference.

**Figure 5:**
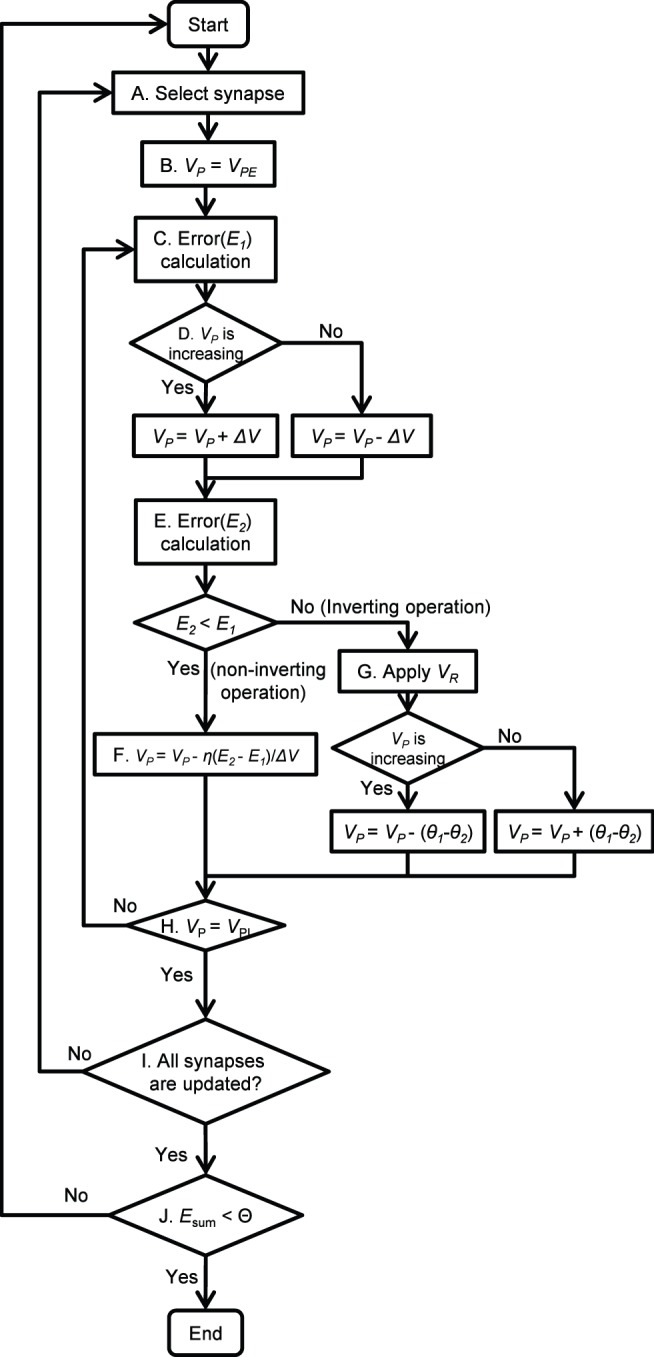
Flowchart of back-propagation learning operation. The write pulses (*V*_P_) for the excitatory and inhibitory synapses are defined as *V*_PE_ and *V*_PI_, respectively. *V*_P_ is slightly changed (*ΔV*) according to the *V*_P_ direction and the error change is calculated. When the error increases (*E*_2_>*E*_1_), after applying *V*_R_ at step G, *V*_P_ is switched and jumps from one curve to another in Figure 4(b). This *V*_P_ jump eliminates the hysteresis of *θ*_1_−*θ*_2_.



Moreover, to eliminate the effect of the hysteresis of the conductance, we applied the following procedures. The detailed flowchart is shown in Figure 5. In this flowchart, *V*_PE_ and *V*_PI_ are *V*_P_s for the excitatory and inhibitory synapses, respectively, whose values are stored in an external memory. *Θ* is the designated threshold value to exit from the learning procedure. *E*_sum_ is the sum of the square errors for all target output values and is calculated according to:

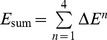
(11)The learning procedures are as follows:

- Select a synapse to update. In this paper, we started from output-layer.
- First, the FeMEMs for excitatory synapses are updated; *V*_P(old)_ = *V*_PE_.
- Error *E*_1_ at a point is calculated according to the outputs from ANN hardware and target output values.
- Slightly larger *V*_P_ in amplitude than the stored previous *V*_P(old)_ is applied to the FeMEM constructing the target synapse. That is, when *ΔV*>0, *V*_P(old)_ + *ΔV* is applied in the case of increasing *V*_P_, and *V*_P(old)_ − *ΔV* is applied in the case of decreasing *V*_P_.
- Error *E*_2_ at a point is calculated in the same manner as step C.
- If *E*_2_≤*E*_1_, according to (10), *V*_P(new)_ is updated and stored in an external memory. In this case, *V*_R_ is not applied. We term this operation “non-inverting operation”.
- If *E*_2_> *E*_1_, *V*_R_ is applied according to *V*_P_ polarity as explained in Figure 4(b). Subsequently, *V*_P_ of the same conductance on another curve in Figure 4(b) is updated and stored; *i.e.*, *V*_P(new)_ = *V*_P_ − (*θ*_1_ − *θ*_2_) if *V*_P_ is increasing and *V*_P(new)_ = *V*_P_ + (*θ*_1_ − *θ*_2_) if *V*_P_ is decreasing. We term this operation “inverting operation”.
- For the FeMEMs for inhibitory synapses, by substituting *V*_P(old)_ to *V*_PI_, *V_P_*s are updated in the same manner as steps C to G.
- Steps A to H are repeated for all synapses in the network.
- If *E_sum_* is larger than *Θ*, then the process returns to step A, else the learning procedure is finished.

At the step G, *V*_P_ is switched and jumps from one curve to another in Figure 4(b). The reset pulse *V*_R_ and this *V*_P_ jump eliminate the hysteresis of *θ*_1_ − *θ*_2_.

## Results and Discussions

### 1. Learning of the boolean logic of exclusive OR

**Figure 6:**
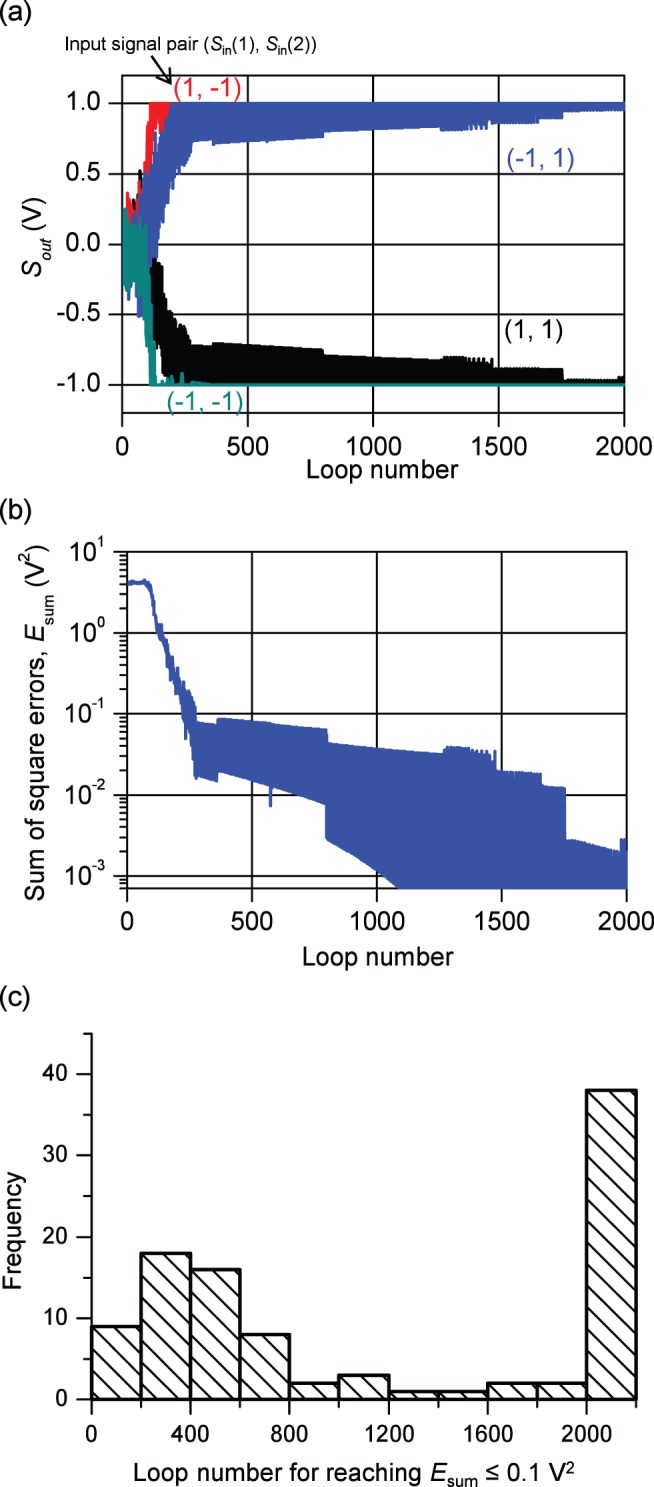
Typical example of the learning operation under the condition of *η* = 0.01 V^−2^. Typical example of the learning operation under the condition of the learning rate (*η*) = 0.01 V^−2^. (a) output from the output-layer neuron (*S*_out_) evolution for four input signal pairs. (b) the sum of the square errors (*E*_sum_) evolution. (c) Histogram of loop number for reaching *E*_sum_≤0.1 V^2^. We denote the probability for reaching *E*_sum_≤0.1 V^2^ within 2000 loops as *P*_C_. In this case, *P*_C_ was about 60%.

To evaluate the proposed learning operation, we numerically analyzed the learning process of the Boolean logic of exclusive OR using (10). The high and low signals were set to 1 and −1 V, respectively. In this analysis, *ΔV* = 10 mV, *η* = 0.01 V^−2^ and *G*_R_ = 10^−5^ S. Initial values of *G*_F_ were randomly set within [0.9×10^−6^, 1.1×10^−6^] S. The learning operation for the output layer was first executed and then the learning operation for the hidden layer. The results are shown in Figure 6. One loop involves the update of all synapses in output and hidden layers.

*S*_out_ values fluctuated until about 200 loops; however, the values gradually became correct afterward (Figure 6(a)). *E*_sum_ gradually decreased from 200 loops, and the learning operation successfully converged (Figure 6(b)). By changing the initial values of *G*_F_ randomly, we simulated 100 learning processes and examined the loop number required for reaching *E*_sum_≤0.1 V^2^. As shown in Figure 6(c), the loop number for reaching *E*_sum_≤0.1 V^2^ with the highest frequency was 200–400 bin. However, there were cases in which *E*_sum_ was larger than 0.1 V^2^ after more than 2000 loops. Here we denote the probability for reaching *E*_sum_≤0.1 V^2^ within 2000 loops as *P*_C_. In this case, *P*_C_ was about 60%.

### 2. Adoption of conductance dispersion

**Figure 7:**
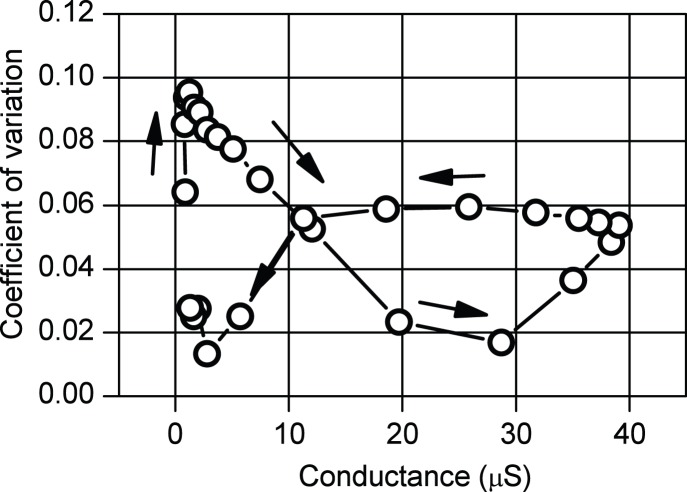
Coefficient of variation of conductance. Coefficient of variation (CV) of conductance is calculated by dividing the standard deviation by the mean. The results show that the CV is less than 0.1 for all values of conductance.

In the Section 4.1, a simulation was carried out for the conductance characteristics of the FeMEM using (6). However, as seen from Figure 4(b), the conductance showed dispersion. In this Section, we adopt this dispersion numerically to simulate under a more realistic condition. From the results in Figure 4(b), the coefficient of variation (CV), which is calculated by dividing the standard deviation by the mean, was plotted as shown in Figure 7.

The results show that the CV is less than 0.1 for all values of conductance. Therefore, when we calculate *G*_F_ for applied *V*_P_, we introduced random dispersion so that the CV = 0.1. The conductance *G*_F_′, which involves the dispersion, is calculated according to
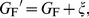
(12)where *ξ* indicates the Gaussian dispersion, which is calculated by Box–Muller method.

Here, we introduce the properties of ferroelectric material to this *ξ*. Under the condition that voltage pulses have the same polarity and sufficient pulse widths, the polarization of the ferroelectric changes only when the maximum amplitude voltage in its history is applied. In this experiment, the pulse width of *V*_P_ was set to 1 ms, which is sufficiently wide because the switching time of this device is less than 1 µs [13].

In the inverting operation, because the polarity changes, *G*_F_′ changes according to (12). However, in the non-inverting operation, the *G*_F_′ changes only when the maximum amplitude voltage is applied according to the properties of ferroelectric. As a result, *G*_F_′ never decreases in cases of *V*_P_>0 and never increases in cases of *V*_P_<0. In the following, we term this “restriction effect”. In the simulation, this effect was realized by setting *ξ* = 0 in the case that (*ξ*<0 and *V*_P_>0) or (*ξ*>0 and *V*_P_<0).

When the error is decreasing (*E*_2_<*E*_1_), because the non-inverting operation is chosen, if *ξ* is not too large, the error hardly increases by *G*_F_’. Needless to say, if *ξ* is too large, because *G*_F_′ jumped over the adequate conductance value, the error increases. It should be noted that the restriction effect enhanced the correct *G*_F_′ change.

**Figure 8:**
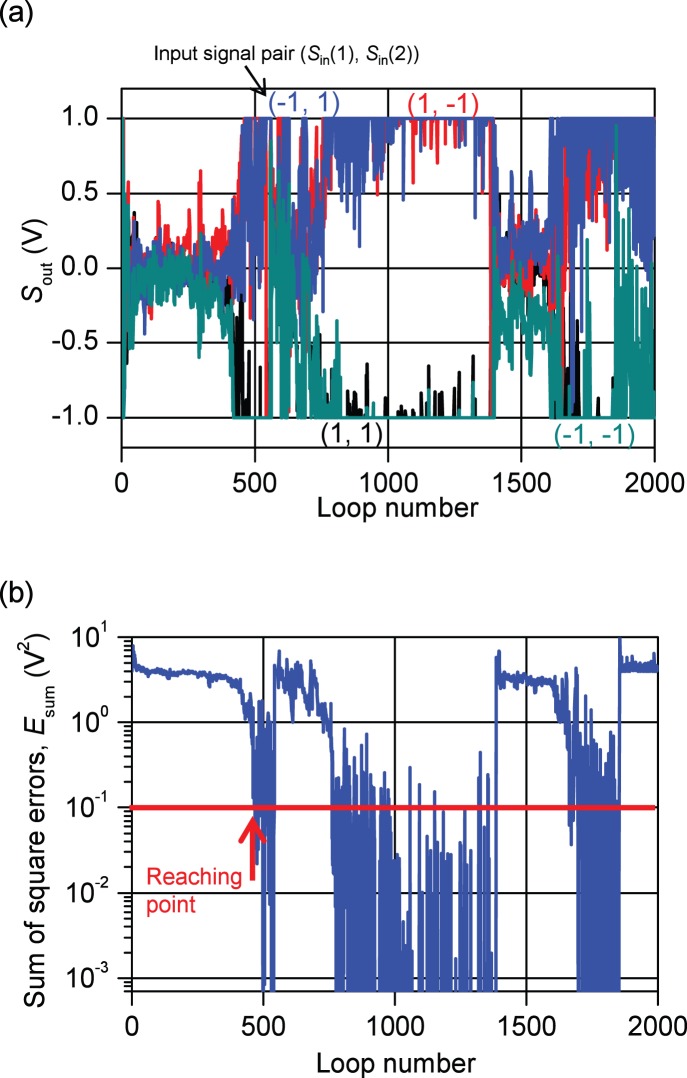
Typical example of the learning operation under the condition of CV = 0.1 and *η* = 0.01 V^−2^. Typical example of the learning operation under the condition of the coefficient of variation (CV) = 0.1 and the learning rate (*η*) = 0.01 V^−2^. (a) output from the output-layer neuron (*S*_out_) evolution for the four input signal pairs. (b) the sum of the square errors (*E*_sum_) evolution. “Reaching point” indicates the loop number at which the *E*_sum_ is smaller than 0.1 V^2^ for the first time in the operation.

Taking this restriction effect into consideration, we simulated the learning process under the condition of CV>0. The simulation results are shown in Figure 8. Though, both *S*_out_ and *E*_sum_ showed large fluctuation, *E*_sum_ rapidly decreased from 400 loops and fallen below the designated value at about 450 loops. In Figure 8(b), this point is indicated as “Reaching point”. However, after that, *E*_sum_ rapidly increased again. These results are very different from those of CV = 0 in Figure 6. When CV = 0, *E*_sum_ decreased gradually from 200 loops and, after that, continued decreasing. When CV = 0.1, *E*_sum_ fluctuated in large scale, however, *E*_sum_ was not always large but fall down again below 10^−3^ V^2^. The results seemed not to diverge.

**Figure 9:**
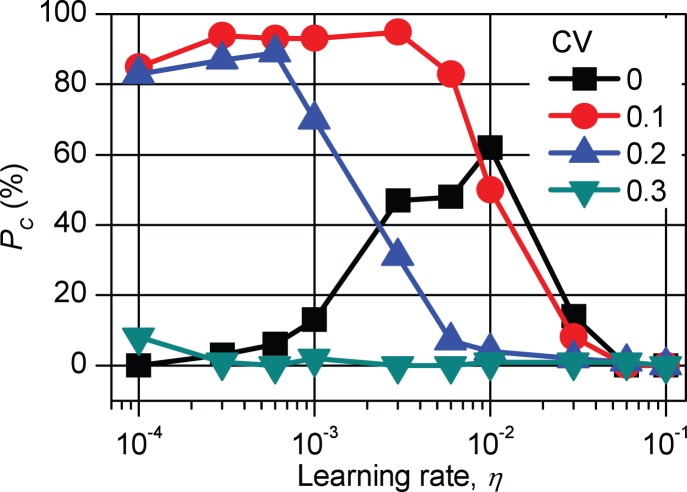
Relation between *P*_C_ and *η* under the condition of coefficient of variation = 0–0.3. *P*_C_ is the probability for reaching sum of the square errors (*E*_sum_) ≤0.1 V^2^ within 2000 loops, and *η* is the learning rate. When the coefficient of variation (CV) is appropriate, the pulse voltage changes adequately even though *η* = 0 V^−2^. These results show that, in wide regions of the CV, high *P*_C_ is realized. When 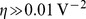, the pulse voltage (*V*_P_) changed so large according to (10) that the conductance of FeMEM (*G*_F_) also changed large and that *P*_C_ became small regardless of CV. When 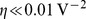, *V*_P_ hardly changed in case of CV = 0. However, in case of CV = 0.1, the learning operation progressed because the *G*_F_’ is not so small according to (12). Moreover, in almost cases, *E*_sum_ was expected to decrease by the restriction effect in non-inverting operation.

Finally, to clarify the effect of the conductance dispersion, we analyzed the relation between *P*_C_ and *η* changing CV values. The results are shown in Figure 9.

When 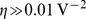, regardless of CV value, because *V*_P_ (and *G*_F_) changed too large according to (12), *G*_F_ jumped over the adequate value. As a result, *P*_C_ became small.

When 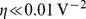, under the condition that CV = 0, *V*_P_ (and *G*_F_) changed so small that *E*_sum_ hardly changed. Consequently, *P*_C_ became small because *P*_C_ is defined as the probability of *E*_sum_≤0.1 V^2^ within 2000 loops. In this case, the maximum *P*_C_ of about 60% was obtained around *η* = 0.01 V^−2^. On the other hand, under the condition of CV>0, though *V*_P_ hardly changed, the learning operation progressed because the *G*_F_′ changed. Moreover, in almost cases, *E*_sum_ was expected to decrease by the restriction effect in case of non-inverting operation. Although it was true that there was a possibility that *E*_sum_ increased in case of inverting operation, it was shown that high *P*_C_ was realized in a large region of *η*≤0.01 V^−2^, especially CV∼0.1. When CV is too large (CV≥0.3), as explained above, because *G*_F_’ jumped over the adequate value, the error increased and *P*_C_ became small regardless of *η* value.

The CV is a difficult parameter to control. When the absolute value of reset voltage (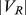) is higher, the repeatability of conductance improves because the ferroelectric polarization is along the major loop, whereas when the 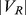 is lower, because the ferroelectric polarization is along the minor loop, the dispersion of conductance increases. Thus, rough control of the CV is possible by changing the reset voltage value. Because *P*_C_ is high in a comparatively wide region of the CV, high *P*_C_ can be achieved by controlling the reset voltage.

As for neural networks, it is commonly known that noise assists in the escape from local minima [24]. Conversely, for analog ANN hardware, noise is harmful to learning because voltages cannot be controlled strictly. As for FeMEM, however, we found that appropriate dispersion realized large *P*_C_ value.

The proposed operation procedure is simple and easy to implement in hardware yet is capable of eliminating the effect of hysteresis and is robust against the dispersion of conductance. Another type of memristor also displays the hysteresis [7], [8]. By expressing the relation between the conductance and the applied voltage as equations and analyzing the CV, our approach can be also applicable to such memristors if they also exhibit restriction effect.

## Conclusions

A BP learning operation was studied for analog artificial neural network (ANN) hardware having a ferroelectric memristor (FeMEM) synapse. The synapse weight was expressed by the channel conductance (*G*_F_) of the FeMEM. After applying a reset pulse, by changing the height of the pulse voltage (*V*_P_), smooth counterclockwise characteristics of *G*_F_–*V*_P_ were observed.

To eliminate the effect of hysteresis of the conductance, we proposed a learning operation, by which *G*_F_ always traveled on either curve of two *G*_F_–*V*_P_ relations. By this operation, because *G*_F_ traveled practically along a continuous function, we confirmed that the simulation of the learning operation converged.

The measured *G*_F_ had a coefficient of variation up to 0.1. Therefore, we adopted conductance dispersion numerically in the simulation. As a result, the dispersion introduced large fluctuation in the converging process; however, the probability (*P*_C_) for reaching *E_sum_*≤0.1 V^2^ within 2000 loops was not so poor. Moreover, when the learning rate was smaller than 0.01, *P*_C_ greatly improved to 85%. These results were obtained by the properties of ferroelectric. When *V*_P_ was not inverted, the dispersion affects only in the direction of decreasing error. Dispersion is not a controllable parameter but a characteristic of the FeMEM; however, it can be roughly changed by the reset voltage.

The proposed operation procedure is simple and easy to implement in hardware. Considering the inevitability of the dispersion of analog circuit elements, this operating procedure is useful for analog ANN hardware. As the scale of ANN processing is increasingly growing, analog ANN hardware is a promising candidate for effective calculation and will play an important role in energy saving for large-scale ANNs.
